# Effects of psilocybin, psychedelic mushroom extract and 5-hydroxytryptophan on brain immediate early gene expression: Interaction with serotonergic receptor modulators

**DOI:** 10.3389/fphar.2024.1391412

**Published:** 2024-04-18

**Authors:** Elad Lerer, Alexander Botvinnik, Orr Shahar, Meitar Grad, Karin Blakolmer, Noam Shomron, Amit Lotan, Bernard Lerer, Tzuri Lifschytz

**Affiliations:** ^1^ Biological Psychiatry Laboratory and Hadassah BrainLabs Center for Psychedelic Research, Hadassah Medical Center, Hebrew University, Jerusalem, Israel; ^2^ Israel Institute for Biological Research, Ness Ziona, Israel; ^3^ Sagol School of Neuroscience, Tel Aviv University, Tel Aviv, Israel; ^4^ Faculty of Medical and Health Sciences, Tel Aviv University, Tel Aviv, Israel; ^5^ Parow Entheobiosciences (ParowBio), Chicago, IL, United States

**Keywords:** psychedelics, psilocybin, 5-HTP, hTR, cfos, Egr1, Egr2

## Abstract

**Background:** Immediate early genes (IEGs) are rapidly activated and initiate diverse cellular processes including neuroplasticity. We report the effect of psilocybin (PSIL), PSIL-containing psychedelic mushroom extract (PME) and 5-hydroxytryptophan (5-HTP) on expression of the IEGs, *cfos, egr1*, and *egr2* in mouse somatosensory cortex (SSC).

**Methods:** In our initial experiment, male C57Bl/6j mice were injected with PSIL 4.4 mg/kg or 5-HTP 200 mg/kg, alone or immediately preceded by serotonergic receptor modulators. IEG mRNA expression 1 hour later was determined by real time qPCR. In a replication study a group of mice treated with PME was added.

**Results:** In our initial experiment, PSIL but not 5-HTP significantly increased expression of all three IEGs. No correlation was observed between the head twitch response (HTR) induced by PSIL and its effect on the IEGs. The serotonergic receptor modulators did not significantly alter PSIL-induced IEG expression, with the exception of the 5-HT2C antagonist (RS102221), which significantly enhanced PSIL-induced egr2 expression. 5-HTP did not affect IEG expression. In our replication experiment, PSIL and PME upregulated levels of *egr1* and *cfos* while the upregulation of *egr2* was not significant.

**Conclusions:** We have shown that PSIL and PME but not 5-HTP (at a dose sufficient to induce HTR), induced a significant increase in *cfos* and *egr1* expression in mouse SSC. Our findings suggest that *egr1* and *cfos* expression may be associated with psychedelic effects.

## 1 Introduction

Immediate early genes (IEGs) are rapidly and transiently activated by extracellular and intracellular stimuli (Bahrami and Drabløs, 2016). Activation of IEGs initiates a series of intracellular events including phosphorylation of key proteins and processes associated with neural plasticity and memory (Minatohara et al., 2016; Gallo et al., 2018). IEGs are essentially activated by changes in neuronal activity underscoring that they are merely a consequence of neural activation (Minatohara et al., 2016). C*fos*, *egr1* and *egr2* are among several IEGs in the central nervous system that are of interest because of their reported role in the effects of psychedelic drugs on neural function (González-Maeso et al., 2003; González-Maeso et al., 2007; Grieco et al., 2022a). C*fos,* a member of the *fos* gene family, is a proto-oncogene that is rapidly induced in response to neuronal activity, acts as a transcription factor and plays a pivotal role in a variety of neural processes including synaptic plasticity. Other IEGs, such as *arc,* are effectors rather than transcription factors (Grieco et al., 2022b). Like *cfos*, *egr1* (*zif268* or *ngfI-a*) encodes a transcription factor that is important in brain development and adult neuronal activity including learning and memory, response to injury and synaptic plasticity (Duclot and Kabbaj, 2017). *Egr2* (*krox20*) is essential for brain development since knockout of this gene is lethal (Duclot and Kabbaj, 2017). In the adult central nervous system, *egr2* is important for myelination and synaptic plasticity (Petazzi et al., 2023). Identifying the specific immediate-early genes (IEGs) activated by psychedelics and their expression patterns could help elucidate the molecular mechanisms and potential therapeutic applications of these compounds.

Gonzalez-Maeso and colleagues (González-Maeso et al., 2003; González-Maeso et al., 2007; de la Fuente Revenga et al., 2021) have reported that *egr1* and *egr2* are specifically activated in somatosensory cortex (SSC) of mice by 5-HT2A receptor agonists that induce a head twitch response (HTR) while 5-HT2A agonists that do not induce HTR activate *cfos* only. HTR is regarded as a rodent correlate of psychedelic activity in humans (Halberstadt et al., 2020). Thus, 2,5-dimethoxy-4-iodoamphetamine (DOI), lysergic acid diethylamide (LSD) and quipazine all induced HTR and significantly activated *cfos*, *egr1* and *egr2* in mouse SSC while lisuride (a 5-HT2A agonist that does not induce HTR) significantly activated *cfos* only (González-Maeso et al., 2003; González-Maeso et al., 2007; de la Fuente Revenga et al., 2021). Although there is evidence that the widely used psychedelic agent, PSIL, has significant effects on the expression of IEGs (e.g., (Jefsen et al., 2021), its effect on *cfos*, *egr1* and *egr2* has not been systematically studied in mice also examined for HTR. Since PSIL induces HTR (Shahar et al., 2022), according to the hypothesis of Gonzalez-Maeso and colleagues, it should significantly activate all three IEGs in mouse SSC. A further key question in this context relates to the effect of 5-hydroxytryptophan (5-HTP), the precursor of serotonin (5-HT), on *cfos*, *egr1* and *egr2* in mouse SSC. While it is well established that 5-HTP induces significant HTR (Corne et al., 1963; Shahar et al., 2022), psychedelic effects of 5-HTP have not been reported.

In the current study, we examined the effect of PSIL on *cfos*, *egr1* and *egr2* expression in the SSC of mice that had been assessed for HTR (Shahar et al., 2022) and also the effect of 5-HTP on the three IEGs. We also tested a relationship between the intensity of HTR induced by PSIL and the intensity of IEG activation. Furthermore, we used pharmacological probes to examine the role of 5-HT2A, 5-HT1A and 5-HT2C receptors in the effect of PSIL and 5-HTP to activate *cfos*, *egr1* and *egr2*. We hypothesized that PSIL would significantly activate *cfos, egr1* and *egr2* in the SSC and that the expression would be associated with the intensity of the HTR response. Furthermore, we hypothesized that serotonergic receptor modulators would affect PSIL-induced IEG expression in the same way as they affected HTR in our previous study (Shahar et al., 2022). To ensure the robustness of our findings, we conducted a replication study to validate the effect of PSIL on IEG expression in which HTR was not measured. In the second experiment, we also examined the effect of psychedelic mushroom extract (PME) which we have recently shown to induce more robust molecular effects than PSIL and differential metabolic effects (Shahar et al., 2024). Our findings indicate that *egr1* and *cfos* expression may be associated with psychedelic effects.

## 2 Materials and methods

### 2.1 Animals

Experiments were performed on adult (12 weeks old) C57BL/6J male mice. Comparisons were made between groups of 8–10 mice in each of the treatment groups. Animals were housed under standardized conditions with a 12-h light/dark cycle, stable temperature (22°C ± 1 °C), controlled humidity (55% ± 10%) and free access to food and water, and were allowed to acclimatize 1 week after arrival in the animal facility. Experiments were conducted in accordance with AAALAC guidelines and were approved by the Authority for Biological and Biomedical Models Hebrew University of Jerusalem, Israel, Animal Care and Use Committee (No. MD-21–16563–4). All efforts were made to minimize animal suffering and the number of animals used.

### 2.2 Drugs

PSIL was supplied by Usona Institute, Madison, WI, USA and was determined by AUC at 269.00 nm (UPLC) to contain 98.75% PSIL. 5-HTP, M100907 and 8-OH-DPAT were purchased from Sigma-Aldrich, Israel. RS-102221 was purchased from Biotest, Israel. PSIL was dissolved in 100% saline (0.9% NaCl) solution. PME was supplied by Back of the Yards Algae Sciences (Chicago, IL, USA), dissolved in 0.9% NaCl and administered intraperitoneally at a PSIL dose of 4.4 mg/kg as described in our recent report (Shahar et al., 2024). 5-HTP was dissolved in 5% DMSO +95% saline (0.9% NaCl) solution. M100907, 8-OH-DPAT, and RS-102221 were dissolved in a 5% DMSO +95% saline (0.9% NaCl) solution. All solutions were prepared to a volume of 10 μL/g. Vehicle-treated condition represents injection of the appropriate solution to the equivalent volume of the drug administered. Drugs were administered by i.p. injection in a standard injection volume of 300 µL immediately before HTR was performed. Modulators were injected immediately before PSIL or 5-HTP. Drug dosages were as follows: PSIL 4.4 mg/kg, 5-HTP 200 mg/kg, M100907 2 mg/kg, 8-OH-DPAT 2 mg/kg, RS-102221 4 mg/kg. The PSIL dose of 4.4.mg/kg was chosen to correlate with a 25–30 mg dose commonly administered in human studies by the use of *DoseCal: a virtual calculator for dosage conversion between the animal species* (https://dosecal.cftri.res.in/) developed by Janhavi et al. (2022) which is based on Nair and Jacob (2016) and uses the formula: human equivalent dose (HED) = animal dose x human Km/animal Km (where Km is body weight divided by body surface area). The 5-HTP dose was chosen on the basis of our prior dose response study of HTR (Shahar et al., 2022). To avoid confounding effects all treatments were administered in a counterbalanced way. Assays were performed with the experimenters blind to treatment assignment. To summarize, the first experiment had two treatment groups - PSIL and 5-HTP and a Vehicle group. All groups were tested both with and without administration of serotonergic modulators beforehand. The second experiment had two treatment groups - PSIL and PME and a Vehicle group, and these groups did not receive any serotonergic modulators prior to testing.

### 2.3 Head twitch response

Head twitch response (HTR) was measured in the first experiment where mice were administered PSIL, for 20 min immediately after injection of the drug at ZT4 (11 a.m.), 4 h after lights on at 7a.m., by means of a magnetometer apparatus. The HTR data was obtained from mice that had undergone prior evaluation of HTR as detailed in our previous study (Shahar et al., 2022). Brain samples were obtained from the same mice 1 hour later at ZT5. HTR experiments were performed in a standard experimental room by the same investigators (AB and OS). HTR was not evaluated in the second experiment where PSIL and PME were administered.

### 2.4 Dissection of SSC

Mice were sacrificed by injection of pental 13.33 mg/kg i.p, brains were rapidly removed and SSCs were bilaterally dissected, 1 hour after drug administration as in the studies of Gonzalez-Maeso and colleagues (González-Maeso et al., 2003; González-Maeso et al., 2007; de la Fuente Revenga et al., 2021). SSC was studied in order to be comparable to this prior work. The coordinates of the tissue dissected were from cortex Bregma 0.38 mm to Bregma −1.82 mm, depth of 0.2 mm–3.5 mm, and laterally from 1.75 mm to 4.25 mm. The weight of each SSC collected ranged between 17 mg and 22 mg. The dissected tissue was submerged in 5 volumes of RNAlater solution (Invitrogen, US), and snap frozen in −80 °C liquid nitrogen before being transferred to a −80 °freezer.

### 2.5 RNA extraction

After thawing on ice, somatosensory cortices were removed from RNAlater solution, homogenized in 1 mL of cold QIAzol Lysis Reagent (Qiagen, Germany) using the TissueLyser II homogenizer (Qiagen, Germany). After incubation at room temperature (RT) for 5 min, 200 μL of chloroform (Bio-Lab Ltd., Israel) was added to each sample, and tubes were shaken manually for 15 s. Following another incubation at RT for 3 min, tubes were centrifuged for 20 min at 4 °C at full speed (13,800 rpm; Eppendorf Centrifuge 5417R; Eppendorf, Germany). Once the mix was separated into three layers, the uppermost RNA-containing clear layer was removed and placed in a fresh tube, to which 1:1 (*v*/*v*) isopropanol (Bio-Lab Ltd., Israel) was added to precipitate the RNA. After briefly shaking the tubes, they were incubated at RT for 5 min, after which they were centrifuged for 15 min at 4 °C at full speed (13,800 rpm). Once the RNA had precipitated, the isopropanol was removed and the pellet was washed twice with 1 mL of 75% ethanol (Bio-Lab Ltd., Israel) mixed with DEPC-treated water (Biological Industries, Israel) and centrifuged for 5 min at 4 °C at full speed. After removal of ethanol, the tubes were left to dry for 15–25 min. Once dry, 20–35 μL of DEPC-treated water was added to each tube. Final RNA concentrations were measured using the Thermo Scientific NanoDrop 2000 spectrophotometer (Thermo Fisher Scientific, USA).

### 2.6 Quantitative reverse transcription polymerase chain reaction (qRT-PCR)

*Reverse transcription*: Extracted total RNA was used as input for mRNA complementary deoxyribonucleic acid (cDNA) synthesis. Reverse transcription (RT) of mRNA was conducted using random primers and the High-Capacity cDNA Reverse Transcription Kit (Thermo Fisher Scientific, USA). The SimpliAmp thermal cycler (Applied Biosystems by Thermo Fisher Scientific, USA) was used under the following conditions: 10 min at 25 °C, 120 min at 37 °C, 5 min at 85 °C, and a final step of 4 °C until the end.

*Real-time quantification*: mRNA expression levels were assessed using the PerfeCTa SYBR Green FastMix (Quantabio, USA), according to the manufacturer’s instructions, using the StepOnePlus Real-Time PCR System (Applied Biosystems by Thermo Fisher Scientific, USA). Thermal cycler conditions were as follows: 20 s at 95 °C, 40 amplification cycles (3 s at 95 °C to denature, and 30 s at 60 °C to anneal and extend), and a melt curve: 15 s at 95 °C to denature, 60 °C for 60 s, and an increase of 0.6 °C every 5 s (including a plate read) until reaching 95 °C. Expression values were calculated based on the comparative cycle threshold (Ct) method. Murine mRNA expression levels were normalized to glyceraldehyde 3-phosphate dehydrogenase (*Gapdh*). mRNA levels are shown as fold change (FC) relative to the control group’s expression levels. Expression profiles (measured as Ct of each sample at a constant fluorescence threshold) of the Gapdh reference gene were consistent across groups, regardless of treatment. This was also validated via one-way ANOVA, which showed no significant differences in Gapdh expression profile across treatments of each experimental group (PSI + VEH, *p* = 0.47; PSI + Modulators, *p* = 0.77; 5-HTP + Modulators, *p* = 0.52). Specific primers for the detection of mRNA expression were ordered from IDT Corporation, Inc. (USA) and diluted to 10 mM in DEPC-treated water according to the manufacturer’s instructions (see Table 1 for specific sequences).

**TABLE 1:** Specific primers for the detection of mRNA expression.

*Gapdh*	For	AGG​TCG​GTG​TGA​ACG​GAT​T
	Rev	TTG​CCG​TGA​GTG​GAG​TCA​TA
*egr1*	For	GAG​CCG​AGC​GAA​CAA​CCC​TA
	Rev	CCA​TCG​CCT​TCT​CAT​TAT​TCA​GAG
*egr2*	For	GCC​TCG​TCG​GTG​ACC​ATC​TT
	Rev	GCA​GAG​ATG​GGA​GCG​AAG​C
*cfos*	For	CCG​ACT​CCT​TCT​CCA​GCA​TG
	Rev	GCT​GGT​GGA​GAT​GGC​TGT​CA

### 2.7 Statistical analysis

The experimental data are expressed as the mean ± standard error of the mean (SEM). The normality of the data was assessed using the Shapiro-Wilk test and the D’Agostino-Pearson omnibus test, using GraphPad Prism. To determine inter group differences, one-way repeated measure analysis of variance (ANOVA) was used. Tukey’s and Dunnett’s Multiple Comparison Tests were used to analyze *post hoc* comparisons. Animals could be excluded from analysis if their results were greater than two standard deviations from the mean. In practice, 3 animals were excluded for this reason in the second experiment (for *egr1*, 1 from the PME group and 1 from the Vehicle group; for *cfos*, 1 from the Vehicle group). GraphPad Prism, version 9.3.1 software was used for all statistical analyses. *p* < 0.05 two tailed was regarded as significant.

## 3 Results

### 3.1 Effect of PSIL, 5-HTP and PME on expression of *egr1*, *egr2* and *cfos* in mouse somatosensory cortex

**FIGURE 1:**
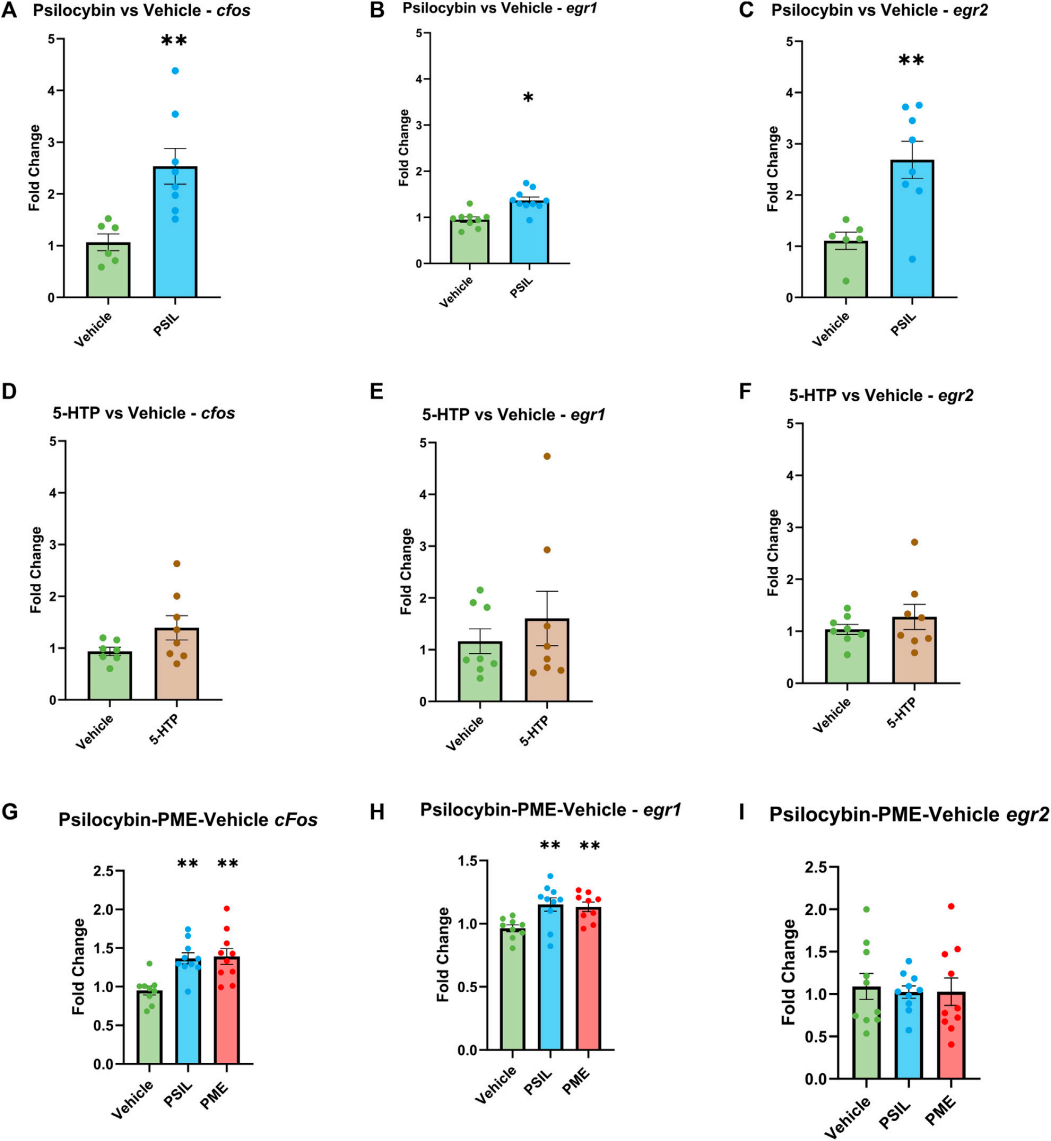
Effect of PSIL **(A–C)** and 5-HTP **(D–F)** on expression of IEGs in mouse SSC. In the first experiment n = six to eight, unpaired *t*-test, compared to Vehicle, *p < 0.05, **p < 0.01. In the second experiment, effect of PSIL and PME **(G–I)**; n = 8–10, one way ANOVA for VEH, PSIL and PME (*p < 0.05, **p < 0.01). Error bars represent SEM.

To test the effect of PSIL and 5-HTP on expression of the three IEGs in the SSC, we performed bilateral comparisons 1 hour after administration. PSIL 4.4 mg/kg i.p (Figure 1, panels 1A- 1C). induced a significant increase in the expression of all three genes (*cfos*, *p* = 0.0048; *egr1*, *p* = 0.042; *egr2*, *p* = 0.0041) while 5-HTP (Figure 1, panels 1D-1F) did not induce a significant increase in expression of any of the genes. In the second experiment (Panels 1G-1I), 1 hour after administration, using one-way ANOVA analysis, both PSIL and PME significantly upregulated *egr1* and *cfos* expression compared to vehicle (F = 6.109, *p* = 0.0069 and F = 8.978, *p* = 0.001, respectively). There was no difference between PSIL and PME effects on *egr1* and *cfos* expression. Neither compound significantly altered *erg2* expression relative to vehicle.

### 3.2 Correlation between IEG expression and HTR

**FIGURE 2:**
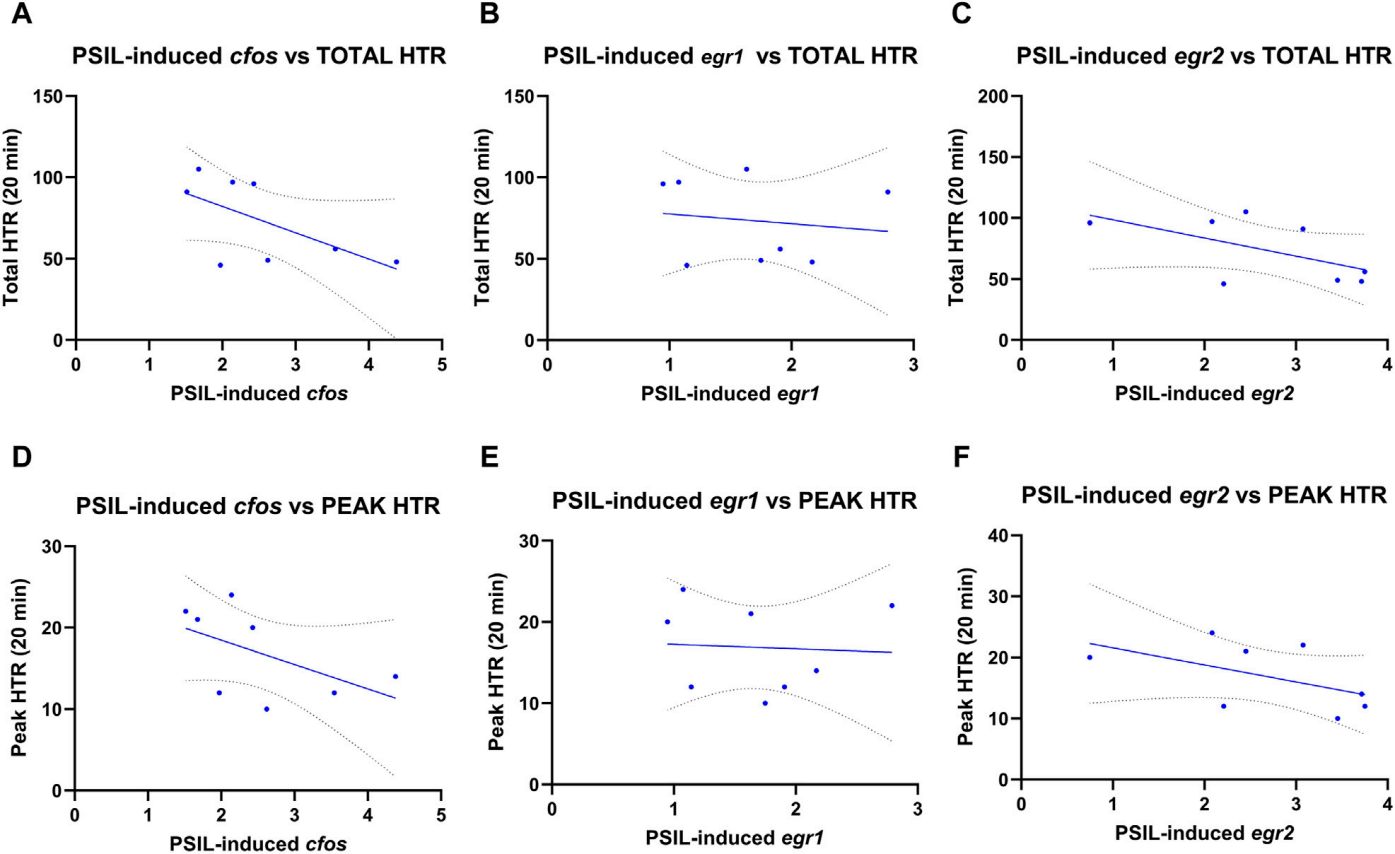
Pearson correlations between cfos, egr1 and egr2 levels induced by PSIL and total **(A–C)** or peak HTR **(D–F)** (n = 8). No significant relationship was observed between HTR and expression of any of the three IEGs.

We examined Pearson correlations between *cfos*, *egr1* and *egr2* levels induced by PSIL and total (Figure 2 panels A–C) and peak HTR (Figure 2 panels D–F) (Shahar et al., 2022). No significant relationship was observed between HTR and expression of any of the three IEGs. HTR was not evaluated in the second experiment.

### 3.3 Effect of 5-HT receptor modulators on PSIL- and 5-HTP-induced induced IEG expression

**FIGURE 3:**
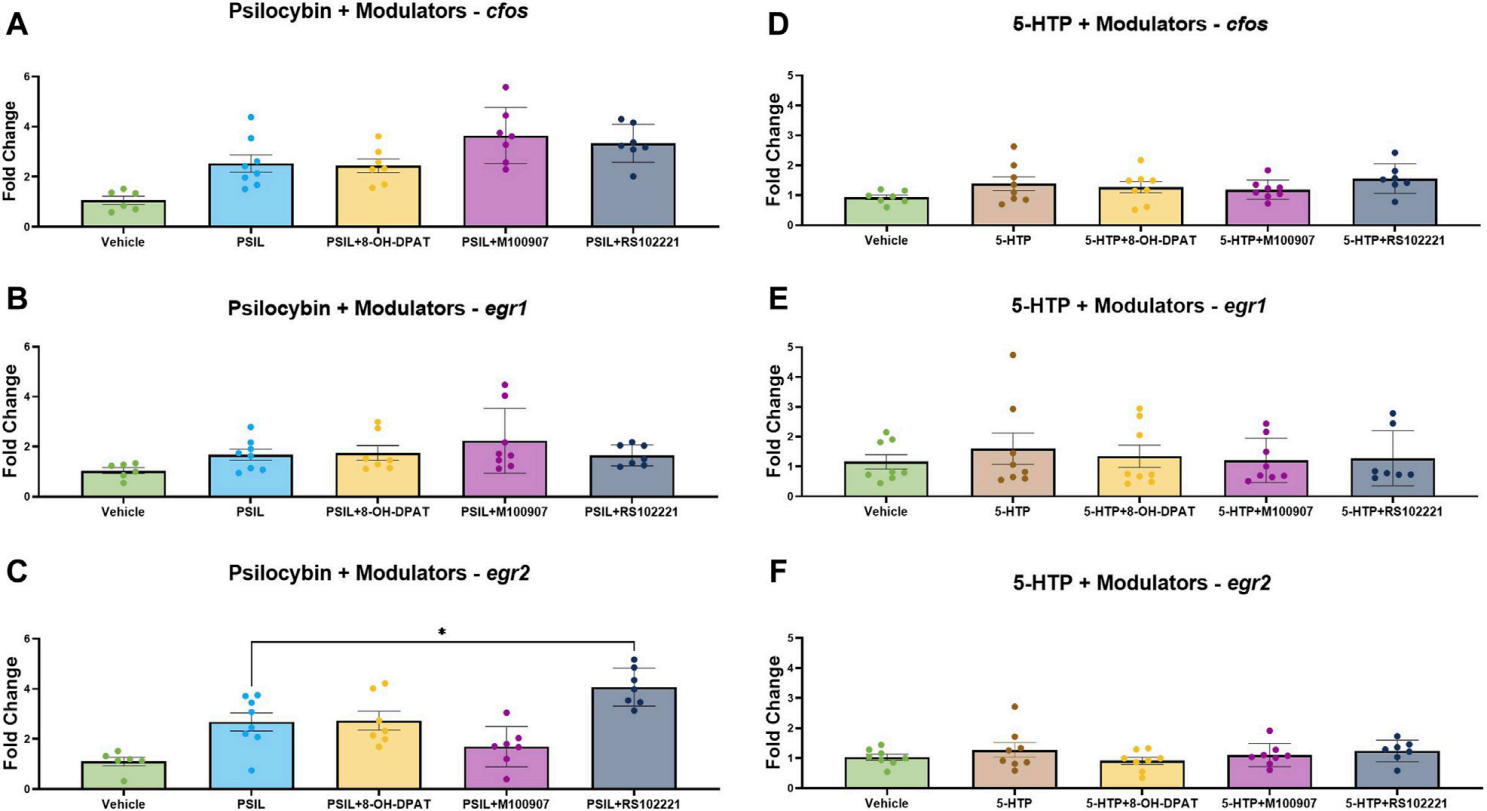
Comparative IEG expression change induced by PSIL **(A–C)** or 5-HTP **(D–F)**, co-administered with a 5-HT receptor modulators (n = 6–8). One-way ANOVA for PSIL-cfos F (4, 30) = 9.008, *p* = 0.0001; PSIL-egr1 F (4, 34) = 0.228, *p* = 0.9203; PSIL-egr2 F (4, 30) = 11.9, *p* < 0.0001. For 5-HTP no ANOVAs were significant. Tukey’s *post hoc*. Test, compared to PSIL, *p < 0.05.

We examined the effect of prior administration 5-HT receptor modulators on the expression of *cfos*, *egr1* and *egr2* induced by PSIL and 5-HTP (Figure 3). One-way analysis of variance (ANOVA) was conducted separately for each IEG tested across all treatment groups: vehicle, psilocybin (PSIL), PSIL + OH-DPAT, PSIL + M100907, and PSIL + RS102221. The ANOVA results were as follows: for the *cfos* gene, F(4, 30) = 9.008, *p* = 0.0001; for the *egr1* gene, F(4, 34) = 0.2288, *p* = 0.9203; and for the *egr2* gene, F(4, 30) = 11.9, *p* < 0.0001. Prior administration of the 5-HT1A agonist, 8-OHDPAT did not prevent the significantly increased expression of *egr1, egr2* and *cfos* induced by PSIL (Figure 3, panels A–C). The 5-HT2A antagonist, M100907, attenuated PSIL-induced *egr2* expression (panel C) but did not alter the increase in *egr1* and *cfos* induced by PSIL (panel A–B). The 5-HT2C antagonist, RS102221, significantly enhanced PSIL-induced *egr2* expression (panel C) but did not significantly alter PSIL-induced *egr1* and *cfos* expression (panel A–B). None of the modulators significantly affected 5-HTP-induced *cfos*, *egr1* and *egr2* expression (Figure 3, panels D, E, F). Examination of 5-HT modulators was not conducted in the second experiment (PSIL and PME).

## 4 Discussion

We observed a significant effect of PSIL and PME administered at a dose of 4.4 mg/kg PSIL 1 hour before, to increase expression of the IEGs *cfos*, *egr1,* in mouse SSC that was replicated for PSIL and an unreplicated effect of PSIL to increase expression of *egr2*. Considered in the context of prior studies, ours is the only one to examine the effect of PSIL and PME on all three IEGs in SSC 1 hour after injection. Jefsen et al. (Jefsen et al., 2021) examined the effect of PSIL (0.5–20 mg/kg) injected to rats 90 min before on 46 target genes and 8 reference genes in rat prefrontal cortex and hippocampus, using real-time quantitative polymerase chain (qPCR) reaction. They found a significant effect of PSIL to increase the expression of *cfos* in prefrontal cortex, a reduction in the expression of *egr2* in the hippocampus and no effect on *egr1*. Other studies reported effects of PSIL on *cfos* expression in mouse brain but did not report effects on *egr1* and *egr2.* Using serial two photon microscopy and light sheet microscopy, Davoudian et al. (Davoudian et al., 2023) found a significant effect of PSIL (1 mg/kg) to increase *cfos* in primary visual cortex, central and basolateral amygdala, and claustrum of mice. They found that select regions exhibited drug-preferential differences with PSIL preferentially increasing *cfos* expression in dorsal raphe, lateral habenula, and insular cortex. Martin and Nichols (Martin and Nichols, 2018) reviewed earlier studies on the effect of psychedelic drugs on IEGs. Most work was done with DOI and effects to increase *cfos* were extensively observed. Effects of PSIL were not reported. Rijsketic et al. (Rijsketic et al., 2023) examined immunofluorescence-labeled, brain-wide *cfos* expression in mice that had been administered PSIL 2 mg/kg 2 hours previously. They found increased *cfos* expression in neocortex, caudoputamen, central amygdala, and parasubthalamic nucleus and decreased *cfos* expression in the hypothalamus, cortical amygdala, striatum, and pallidum. In comparing our findings to these prior studies it is noteworthy that these authors used lower doses of PSIL than we did, except for Jefsen et al. (Jefsen et al., 2021) who examined a range of doses up to 20 mg/kg and all these authors examined later time points following injection than we did (90 min–3.5 h). Wenthur et al. (Jones et al., 2023) studied psilocybin’s pharmacokinetics and effects in mice, finding half-lives of 8 ± 1 min for psilocybin and 35 ± 4 min for its active metabolite psilocin after intraperitoneal administration. In our recent study comparing chemically synthesized psilocybin (PSIL) and psychedelic mushroom extract (PME) effects on mouse brain, we measured plasma psilocin levels to ensure differences were not due to psilocin variations. Plasma psilocin levels after 4.4 mg/kg i.p. PSIL or PME were similar at 15, 30, and 60 min, with no statistically significant differences.

No previous studies have examined the relationship between HTR and gene expression in mice administered psychedelic compounds. In the current study, we compared our gene expression data with the HTR data from our previous study (Shahar et al., 2022), which used the same mice as those employed in the present experiment. We did so, examining HTR immediately following injection of PSIL for 20 min duration and IEG expression 40 min later (60 min after PSIL injection). We found no significant relationship suggesting that the intensity of PSIL-induced HTR is not reflected in the degree to which IEGs are activated. If this observation holds true for other psychedelic drugs it would suggest that initial and possibly downstream cellular effects of psychedelic compounds are not directly related to their psychedelic effects, if these are indeed reflected by their effects on HTR as suggested (Halberstadt et al., 2020). However, it should be noted that the correlation effects we observed are limited by sample size and require further examination in larger groups of mice of different ages and also in females as well as males and using additional PSIL doses. Furthermore, it is thought that 5-HT2A receptors in the prefrontal cortex play a key role generation of the head twitch response (Willins and Meltzer, 1997). The role of the somatosensory cortex (SSC) has not been studied specifically. Thus, it is possible that the SSC may not be directly involved in HTR generation in which case a correlation between HTR and IEG expression in the SSC would not be observed. We did not examine the relationship between 5-HTP and PME effects (as well as PSIL in the second experiment) on HTR and IEGs since HTR was not measured in the same mice.

While we have shown that PSIL induced a significant increase in *egr1* and *cfos* in mouse SSC, consistent with its effect to increase HTR in the same mice as reported in our previous study (Shahar et al., 2022), the effects of 5-HT receptor modulators on PSIL-induced changes in gene expression diverged from their effects on HTR (Shahar et al., 2022). Thus, the 5-HT1A agonist, 8-OH-DPAT, inhibited PSIL-induced HTR (Shahar et al., 2022) but it did not alter the effect of PSIL on IEGs. This difference could potentially be attributed to a disparity in the mechanisms of the two phenomena that is reflected in the lack of correlation between PSIL-induced HTR and PSIL-induced IEG expression. It should be emphasized in this context that the mechanism whereby 5-HT1A agonists attenuate HTR induced by PSIL is not known. The 5-HT2A receptor antagonist, M100907, blocked PSIL-induced HTR but surprisingly, did not significantly alter PSIL effects on *cfos*, *egr1* and *egr2*, despite their primary activation via the 5-HT2A receptor (González-Maeso et al., 2003; González-Maeso et al., 2007), that indicating other pathways may be involved in the regulation of IEGs by PSIL. The 5-HT2C antagonist, RS102221, significantly enhanced PSIL-induced *egr2* expression and increased HTR; however, the effect of PSIL to increase egr2 was not internally replicated. These findings suggest that serotonergic receptor mechanisms implicated in the effect of PSIL on IEGs overlap only partially, if at all, with their role in PSIL-induced HTR, as reported in our previous study. The present study aimed to elucidate the direct effects of psychedelics on immediate early gene (IEG) expression in relation to the HTR, while noting the potential value in exploring how antidepressant-like behavior correlates with IEG expression. It is important to acknowledge that the current study is focused on the somatosensory cortex (SSC), thus may not fully encompass all brain regions implicated in the effects of the tested compounds. The changes in IEGs expression within the SSC could potentially represent downstream effects resulting from the activation of other brain areas. To gain a more comprehensive understanding of the neural mechanisms underlying the actions of psychedelics, future investigations should extend the analysis of IEG expression to additional brain regions such as the prefrontal cortex.

In contrast to the effect of PSIL, 5-HTP, at a dose sufficient to induce significant HTR (Shahar et al., 2022), did not affect IEG expression. Gonzalez-Maeso and colleagues (González-Maeso et al., 2003; González-Maeso et al., 2007; de la Fuente Revenga et al., 2021) have suggested that *egr1* and *egr2* expression are correlated with the effect of 5-HT2A agonists to induce HTR. The absence of an effect of an HTR-inducing dose of 5-HTP to increase IEG expression is not consistent with this suggestion. It should be noted, however, that 5-HTP has not been reported to be psychedelic in humans. It may thus be speculated that effects of 5-HT2A agonists on *egr1* and possibly *egr2* expression may reflect a correlation with potential to induce psychedelic effects rather than an effect on HTR, an interesting possibility that is worthy of further investigation.

## 5 Conclusion

We have found that PSIL and PME induce a significant increase in the IEGs *cfos*, *egr1* and possibly *egr2* in mouse SSC 1 hour after administration. This effect is not correlated with the effect of PSIL on HTR. Furthermore, the effects of serotonin receptor modulators on PSIL induced changes in IEG expression are not consistent with their effects on PSIL induced HTR. Since IEG activation initiates downstream molecular and functional effects, these findings suggest that the cellular effects of PSIL may not be correlated with its psychedelic effects. Of further note in this context is that 5-HTP, which has not been reported to induce psychedelic effects in humans although it does induce HTR in rodents, did not significantly alter IEG expression. Further studies are needed to explore this key interface between behavioral and molecular effects of psychedelics.

## Data Availability

The original contributions presented in the study are included in the article/Supplementary Material, further inquiries can be directed to the corresponding authors.
